# An Improved Real Time Image Detection System for Elephant Intrusion along the Forest Border Areas

**DOI:** 10.1155/2014/393958

**Published:** 2014-01-20

**Authors:** S. J. Sugumar, R. Jayaparvathy

**Affiliations:** ^1^Department of Electrical and Electronics Engineering, Coimbatore Institute of Technology, Avinashi Road, Civil Aerodrome PO, Coimbatore, Tamil Nadu 641 014, India; ^2^Department of Electronics and Communication Engineering, SSN College of Engineering, Kalavakkam, Old Mahabalipuram Road, Chennai, Tamil Nadu 603 110, India

## Abstract

Human-elephant conflict is a major problem leading to crop damage, human death and injuries caused by elephants, and elephants being killed by humans. In this paper, we propose an automated unsupervised elephant image detection system (EIDS) as a solution to human-elephant conflict in the context of elephant conservation. The elephant's image is captured in the forest border areas and is sent to a base station via an RF network. The received image is decomposed using Haar wavelet to obtain multilevel wavelet coefficients, with which we perform image feature extraction and similarity match between the elephant query image and the database image using image vision algorithms. A GSM message is sent to the forest officials indicating that an elephant has been detected in the forest border and is approaching human habitat. We propose an optimized distance metric to improve the image retrieval time from the database. We compare the optimized distance metric with the popular Euclidean and Manhattan distance methods. The proposed optimized distance metric retrieves more images with lesser retrieval time than the other distance metrics which makes the optimized distance method more efficient and reliable.

## 1. Introduction

The Asian elephant (*Elephas maximus*) is highly threatened by habitat fragmentation, habitat loss, and human-elephant conflict. India hosts 60% of Asian elephant population, nearly two-thirds of the elephant population lives either close to or within human-dominated landscapes. Southern India harbors half of India's elephant population containing about 6300 elephants [1]. The increase in human population in India propelled by agricultural and industrial growth has led to the conversion of the forest lands into human settlements. Due to this, the wild elephant and other animal populations face acute shortage of resources such as water and food, making them move often into the human habitat. Hence, there has been severe man-elephant conflict. The conflict has been on the rise in the forest border areas with herds of wild pachyderms straying into human habitation [2]. The surveillance and tracking of these herds are difficult due to their size and nature of movement. The time to recover from the danger is negligible; hence, the loss due to destruction in the farms is more. The elephants are also subject to attack by humans resulting in danger to the life of elephants. According to the authors in [3], poaching for ivory had indeed become a threat with 100–150 tuskers being lost annually to illegal killings.

Human-elephant conflict (HEC) is a key example of the growing competition between people and wildlife for space and resources throughout Africa and Asia. This study explores the correlation of reported HEC incidents within 58 villages between 80 km from the boundary of Kallar to Walayar, Coimbatore, Tamil Nadu, India. Habitat loss and fragmentation is the biggest threat to the continuing survival of Asian elephants in this region. In addition to food crops, forests are being logged for their timber or cleared to make space for cash crop plantations such as rubber, tea, and palm. As the human population has inexorably risen, the forest wild lands in which elephants live have been disappearing. Human-elephant conflict is on the rise and it is a battle that the elephant is losing. As elephant habitat diminishes, the elephants are pushed into increasingly smaller areas. This increases the population density to beyond sustainable levels and food availability grows short. The shortage of fodder has a negative impact on rates of reproduction; hence, normal birth rates begin to decrease. The serious consequence of the shortage of wild food leads to a corresponding increase of crop raiding and incidents of human-elephant conflict [3].

Human-elephant conflict is a rapidly expanding area of research, with conservationists working hard to understand the circumstances under which tensions are the highest between humans and elephants. A number of factors contribute to such conflicts, including population density of humans, elephant habitat structure, weather, time of year, and animal life [4]. A study made in the region of interest shows that elephants move into human habitation due to many reasons.

- Fences and trenches compromised by people who need access to forests.
- Farm lands may funnel them to unprotected adjacent villages.
- Badly planned barriers that do not take elephant behavior into consideration.
- Denying elephant access to a critical water source or foraging area.
- Human activities create abundant secondary vegetation that brings elephants closer to human settlements.
- Artificially maintained water sources attract elephants during drought.
- Traditional migration routes severed by human intervention (e.g., canals, power installations, and cattle fences).

The obvious conclusion to be drawn is that there is no single cause or explanation to account for human-elephant conflict; situations are circumstantial and complex. Rather, elephants and agriculture mix in numerous ways with varying consequences. Human population growth and land occupation for settlement may heighten conflict with elephants. However, it is generally the borders of forests that are the focal points of conflicts. Minimizing human-elephant conflict to reduce the risk of life of both human beings and elephants is of utmost importance. Elephant conservation issues can be divided into two distinct categories:

1. activities that affect elephants directly such as hunting/poaching and capture;
2. developmental activities and human activities leading either to the loss of elephant habitat or its qualitative degradation.

Many methods are followed to avoid HEC. Construction of elephant proof trenches is being done all over the world. In [5], Fernando et al. discussed solar fencing to avoid elephant-human conflict. In [6], King et al. presented the concept of using beehives to mitigate elephant crop depredation. In [7], Loarie et al. discussed about the role of the artificial water sources which allow elephants to reside in forests during dry seasons. In [8], the authors discussed the potential use of satellite technology for conflict mitigation. The elephants tagged with radio collars react violently and damage it and even the elephants die. In [9], Venter and Hanekom proposed the possibility of using the elephant-elephant communication (elephant rumbles) to detect the presence of a herd of elephants in close proximity, In this work, the authors have recorded the low frequency infrasound pattern, but they do not compare with that of other animals to confirm an elephant occurrence. In [10], Vermeulen et al. proposed unmanned aircraft system to survey elephants, in which the elephant images are acquired at a height of 100 m but the small flight time and being expensive do not make it viable. In [11], Dabarera and Rodrigo proposed appearance based recognition algorithms for identification of elephants. Given the frontal face image of an elephant, the system searches the individual elephant using vision algorithms and gives the result as, already identified elephant, or as a new identification. In [12], Ardovini et al. present an elephant photo identification system based on the shape comparison of the nicks characterizing the elephant's ears. In [13], Goswami et al. addressed identifying elephants from photographs, and comparing resultant capture recapture-based population parameter estimates using supervised visual identification of individual variations in tusk, ear fold and lobe shape. The authors show that this is a reliable technique for individual identification and subsequent estimation of population parameters. But in real time, the capture of elephant's front image is not possible.

It is easier to chase elephants before they enter fields and therefore most damage can be averted [3]. Guarding from watch towers, patrolling, and trip wire alarms provide farmers with advance warning of approaching elephants. Once the animals are detected, active crop guarding devices using light and noise are deployed to chase them away. An early warning system to minimize the human-elephant conflict in the forest border areas is proposed in this paper. The system helps mitigate such conflicts in two ways:

- providing warning to people about the anticipated entry of elephants into human habitation;
- providing advance information to the authorities to take action to chase the pachyderms back to the forest.

An early warning system to minimize the human-elephant conflict in the forest border areas using image processing is proposed in this paper. The system helps to detect the elephants even in the presence of other wild animals like Bison, Tiger, and Deer, and so forth. The system also identifies the elephants coming in groups. The reliability of elephant detection is tested and the time to detect the elephant images is optimized with the proposed optimized distance metric.

## 2. Study Area

**Figure 1:**
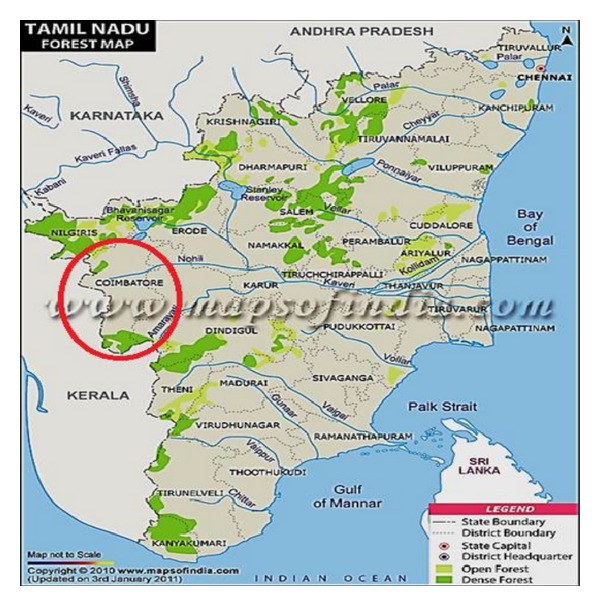
Map showing study area of the Coimbatore district in Tamil Nadu.

Studies were made in the Coimbatore Forest Division, Tamil Nadu, India, as shown in Figure 1. The data were collected from the Coimbatore Forest Department website and interviews with village people affected by elephants and relevant literature. Coimbatore district is richly endowed with hills, forests, rivers, and wildlife. Geographical area of the district is 74,433.72 sq. km with a forest area of 693.48 sq. km (9.33%). The forest of Coimbatore district is divided into two divisions.

South of Palghat Gap lies in the Anamalai Wildlife Sanctuary, which has been designated as a Tiger Reserve in 2008. North of Palghat lies in the Coimbatore forest division. This division is bounded in the north and northwest by Sathyamangalam, Erode, Nilgiris North, and Nilgiris South forest divisions and in the west and southwest by Palghat forest division of Kerala. Coimbatore Forest Division is spread over 693.48 sq. km in six ranges, of which 400 sq. km is conflict prone. The division has 58 villages and 315 km of forest boundary.

**Figure 2:**
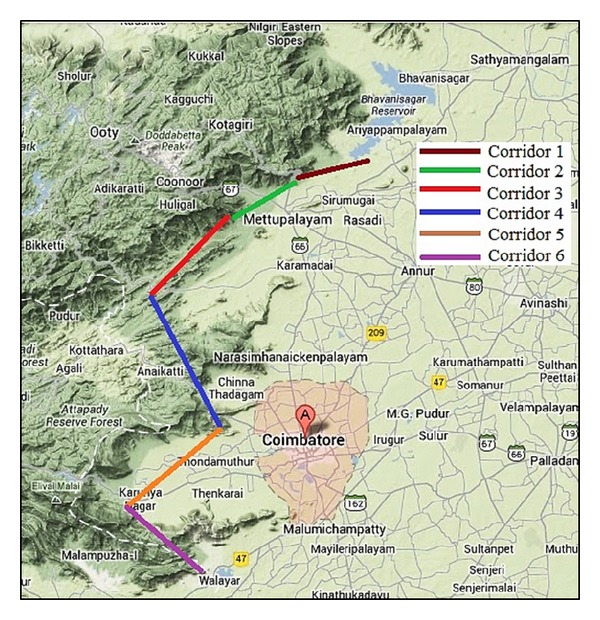
Corridors used by Elephants in the forest border area of Coimbatore.

Six elephant corridors within the Coimbatore forest division are shown in Figure 2. Namely, Jaccanari-Vedar Colony (Corridor 1) in which the length of the corridor is about 12 km and width ranges from 0.2 to 1.5 km. In Kallar-Jaccanari (Corridor 2), the length of the corridor is 7 km and width ranges from 0.2 to 1.5 km. In Kallar-Nellithurai (Corridor 3), the length of the corridor is 10.8 km and width ranges from 0.25 to 3 km. In Anaikatti-Veerapandi (Corridor 4), the length of the corridor is 21 km and effective width ranges from 0.1 to 1.5 km. In Maruthamalai-Thanikandy (Corridor 5), the length of the corridor is 13 km and effective width ranges from 0.4 to 1.5 km. And in Kalkothi-Walayar (Corridor 6), the length of the corridor is 21 kms the effective width ranges from 0.2 to 0.9 km.

In total, there are 85 kms of elephant corridor in the district needs to be protected from human-elephant conflict. The region is also a part of the crucial elephant corridor in this region [4] amounting to a total of 691–914 elephants found in this region. The elephant is one of the most conflict-prone wildlife species in India, causing large-scale damage to crops and human lives. Each year, nearly 400 people and 100 elephants are killed in conflict related instances in India, and nearly 500,000 families are affected by crop damage. Several reasons including habitat fragmentation, degradation of habitat quality, loss of forest cover, laxity in management of physical barriers, and other causes have been cited for the human-elephant conflict (HEC) in the country.

**Figure 3:**
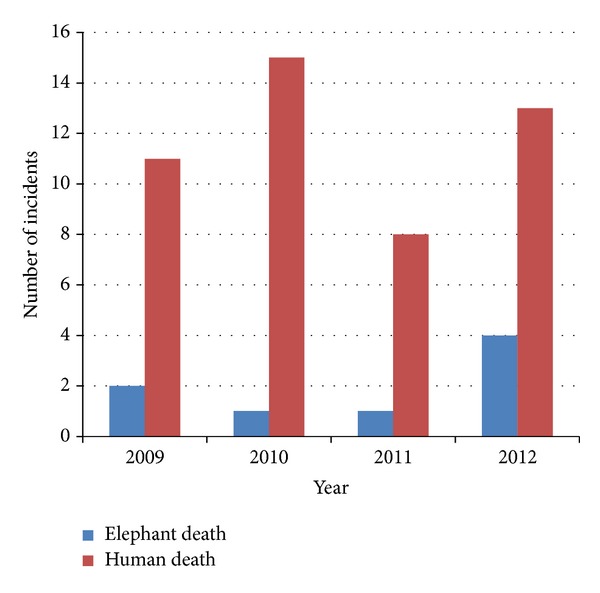
Human and elephant loss in Coimbatore district.

The human habitats bordering the forests around Coimbatore city in south western India are seeing severe human-elephant conflict as the expanding human population propelled by industrial and agricultural growth is increasingly fragmenting elephant habitat in this landscape. The number of incidents of elephants straying into farm lands was 680 in 2011, 844 in 2010, and 560 in 2009. The number of people killed in elephant attacks in Coimbatore was 13 in 2012, 8 in 2011, 15 in 2010, and 11 in 2009. The number of elephants killed by such conflicts was 4 in 2012, 1 in 2011, 1 in 2010 and 2 in 2009 as shown in Figure 3 (Courtesy: the Hindu, Coimbatore, February 5, 2013).

In this region, train hit accidents occur frequently when the elephants try to pass the rail track Walayar, Coimbatore, railway section which is on the forest border area. Thus it has resulted in the death of 20 elephants in the last five years. However, it is generally the edges of forest that are to be protected as those are the focal points of conflict.

Humans go into the forest to graze cattle in day time and guard crops at night and therefore run a higher risk of being killed by elephants. They also try to ride elephants by throwing stones and sticks for which the elephants react hard and even kill the humans. Elephants that wait near villages for nightfall to eat crops have also been known to kill people [3]. During the period 1999–2011, there had been 1,822 incidents of crop damage and 53 cases of property damage caused by elephants and the compensation disbursed was Rs. 2.19 crore.

## 3. Material and Methods

In our previous work [2], seismic geophones used as sensors are buried under the ground to detect the movements of elephants in forest border areas. Elephants walking in the sensing range of the geophones produce vibration which is converted to an electrical signal, processed in an embedded controller and an SMS is sent to the forest officials for necessary action. In such intrusion detection systems, there is a possibility of insufficient vibrations sensed by the geophones as a consequence of weather conditions like rain and soil moisture. Due to this, it may miss the event, that is, elephant movement. An image processing based approach is developed as a solution to the above said problem to identify an intruding elephant in human living areas. The elephant comes out of the forest through certain pockets to enter into human living areas for getting food and water. The cameras mounted on towers or trees capture the image of the intruding elephant which is sent to the base station using RF network. The received image is processed in a PC at the base station and is compared with the stored database image of elephants. The snapshot from the video is taken every 5 seconds and compared with the database image. This image is also updated in database and added. On an image match, an SMS is sent to the forest officials through the GSM transceiver connected with the PC. The hardware setup consists of wireless camera, PIR sensor with signal control module, Atmega microcontroller, GSM module, and the power supply. PIR motion sensor detects the movement and it switches the camera to the capture mode; the camera captures the image over a 20-meter distance. The whole hardware setup is shielded with metal cast to protect from the rain. The elephant pockets in the corridors are identified and these setups are installed to monitor the movement of these herds.

### 3.1. Elephant Image Detection System

Elephant Image Detection System (EIDS) algorithm is developed in this work. A database of 114 images is created by capturing 2 elephants in different postures. Images of elephants in the Sadivayal elephant camp in Coimbatore, South India, have been taken and used in this work. The elephant database images are feature-extracted using Haar wavelet technique and clustered into groups by using *K*-means clustering. A similarity comparison is made by determining the number of significant coefficients in common between the query signature and the signatures of the database using *F* Norm theory. The searched elephant images are then arranged according to the similarity value obtained in a decreasing order. If the matched images are more than 5, an elephant detected message is sent through the GSM to the mobile phone numbers stored in the system. Once the procedure is complete, the system captures the next image and performs the same steps to detect elephants. The Haar wavelet decomposition of elephant image in RGB color space is represented at multiple scales [14]. The Haar wavelet decomposition is computed by iterating difference *d*_*i*_ and average *a*_*i*_ between odd and even samples *s*_*i*_ of the elephant image. Averaging and differencing the elephant image elements are done as follows:

(1) $$\begin{array}{c} a_{i}=\frac{s_{i}+s_{i+1}}{2},\;\;d_{i}=\frac{s_{i}-s_{i+1}}{2}. \end{array}$$

If an elephant image data set *S*_1_, *S*_2_,…, *S*_*N*−1_ contains *N* elements, there will be *N*/2 averages and *N*/2 wavelet coefficient values [15]. The averages are stored in the upper half of the *N* element array and the difference coefficients are stored in the lower half of the array. The averages become the input for the next step in the wavelet computation, for iteration *i* + 1, *N*_*i*_ = *N*_*i*/2_. The recursive iterations continue until a single average and a single difference are calculated [16]. The scaling and wavelet values are represented by *h*_*i*_ and *g*_*i*_, respectively, and are given in (2) and (3). The values of scaling coefficients are given as

(2) $$\begin{array}{c} h_{0}=0.5,\;\;h_{1}=0.5, \end{array}$$

and the values of wavelet coefficients are given as

(3) $$\begin{array}{c} g_{0}=0.5,\;\;g_{1}=-0.5. \end{array}$$

The Haar transform is shown in matrix form as follows:

(4) h0h100⋯g0g100⋯00h0h1⋯00g0g1⋯

The Haar transform for an eight element signal is shown in the following equation. Here, the signal is multiplied by the forward transform matrix *A*:

(5) $$\begin{array}{c} \left[\begin{array}{c} a_{0}\\ a_{1}\\ a_{2}\\ a_{3}\\ d_{0}\\ d_{1}\\ d_{2}\\ d_{3} \end{array}\right]=A\cdot \left[\begin{array}{c} s_{0}\\ s_{1}\\ s_{2}\\ s_{3}\\ s_{4}\\ s_{5}\\ s_{6}\\ s_{7} \end{array}\right], \end{array}$$

where

(6) $$\begin{array}{c} A=\left[\begin{array}{cccccccc} 0.5 & 0.5 & 0 & 0 & 0 & 0 & 0 & 0\\ 0.5 & -0.5 & 0 & 0 & 0 & 0 & 0 & 0\\ 0 & 0 & 0.5 & 0.5 & 0 & 0 & 0 & 0\\ 0 & 0 & 0.5 & -0.5 & 0 & 0 & 0 & 0\\ 0 & 0 & 0 & 0 & 0.5 & 0.5 & 0 & 0\\ 0 & 0 & 0 & 0 & 0.5 & -0.5 & 0 & 0\\ 0 & 0 & 0 & 0 & 0 & 0 & 0.5 & 0.5\\ 0 & 0 & 0 & 0 & 0 & 0 & 0 & -0.5 \end{array}\right]. \end{array}$$

Since the columns of the *A*_*i*_'s are orthogonal to each other, each of these matrices is invertible with respect to *A*_*i*_. The elephant database images are decomposed into multilevel coefficients from −1 to −*J* levels. After decomposition, feature vectors for all the elephant images in the database are obtained using *F*-norm theory [17] as given in (7) and (9). Every image is considered as a square matrix. *A* is a square matrix and *A*_*i*_ is its *i*th-order submatrix. The *F*-norm of *A*_*i*_ is given as

(7) $$\begin{array}{c} {||A_{i}||}_{F}={\left[\overset{i}{\underset{K=1}{\sum }}\;\overset{i}{\underset{I=1}{\sum }}{\left|a_{KI}\right|}^{2}\right]}^{1/2}. \end{array}$$

Let

(8) $$\begin{array}{c} \Delta A_{i}={||A_{i}||}_{F}-{||A_{i-1}||}_{F},\;\;{||A_{0}||}_{F}=0. \end{array}$$

The feature vector of *A* is defined as

(9) $$\begin{array}{c} V_{AF}=\left\{\Delta A_{1},\Delta A_{2},\ldots ,\Delta A_{n}\right\}. \end{array}$$

Vector elements in the feature vector are represented by Δ*A*_*i*_ and Δ*B*_*i*_. The similarity between the two images is given by the following similarity criteria [16]. Let *α*_*i*_ be the similarity of Δ*A*_*i*_ and Δ*B*_*i*_ as follows:

(10) $$\begin{array}{c} \alpha_{i}=\{\begin{array}{cc} \frac{\min\left(\Delta A_{i},\Delta B_{i}\right)}{\max\left(\Delta A_{i},\Delta B_{i}\right)}, & \Delta A_{i}\neq 0\;\;\text{or}\;\;\Delta B_{i}\neq 0,\\ 1, & \Delta A_{i}=\Delta B_{i}=0. \end{array} \end{array}$$

Similarity between the two images lies in between 0 ≤ *α* ≤ 1. The images in the database are arranged according to the similarity match with the query image.

### 3.2. *K*-Means Clustering Algorithm

Clustering is a process of grouping the similar objects from a given data set. The most popular and reliable clustering algorithm is the *K* means clustering algorithm that classifies the input data points into multiple classes based on their inherent distance from each other. Let *S* = {*S*_*i*_,  *i* = 1,2,…, *N*} be the *n*-dimensional data points to be clustered into a set of *K*-clusters, *C* = {*C*_1_, *C*_2_, *C*_3_,…, *C*_*K*_} [18] from the given elephant data set *X* = {*x*_1_,…, *x*_*N*_}, *x*_*n*_ ∈ *E* ^*d*^. The *M*-clustering problem aims at partitioning the elephant data set into *M* disjoint subsets (clusters) *C*_1_,…, *C*_*M*_. The most widely used clustering criterion is the Euclidean distance [19]. Based on this criterion, the clustering of elephant images is grouped depending on the cluster centers *m*_1_,…, *m*_*M*_ as given below

(11) $$\begin{array}{c} E\left(m_{1},\ldots ,m_{M}\right)=\overset{M}{\underset{i=1}{\sum }}\;\overset{M}{\underset{k=1}{\sum }}I\left(x_{i}\in C_{k}\right){||x_{i}-m_{k}||}^{2}, \end{array}$$

where *I*(*X*) = 1 if *X* is true and 0 otherwise.

### 3.3. Proposed Optimized Distance Metric

In this paper, a novel distance metric called optimized distance measure integrated with *K*-means clustering algorithm to improve retrieval time is proposed. We have used the distance metrics in the work for (i) finding similarity between two images and (ii) ordering a set of images based on their distances from a given image. In many image retrieval systems, Euclidean [18] and Manhattan [20] are the popular distance measure algorithms used. We carried out a study on the above two similarity measures and proposed a new distance method called optimized distance measure. The proposed method retrieved more images with faster retrieval rate than the other two methods. The Euclidean distance measures [18] are suitable for the correlation between quantitative and continuous variables and are not suitable for ordinal data and it is given as

(12) $$\begin{array}{c} D=\sqrt{{\left(R_{c}-R_{g}\right)}^{2}+{\left(G_{c}-G_{g}\right)}^{2}+{\left(B_{c}-B_{g}\right)}^{2}}, \end{array}$$

where (*R*_*c*_, *G*_*c*_, *B*_*c*_) are centroids and (*R*_*g*_, *G*_*g*_, *B*_*g*_) are the pixel points or data points. Most of the time is spent to calculate the square root, so it is basically time consuming. The Manhattan distance is the absolute sum of the horizontal and vertical components of the image data matrix. This is essentially a consequence of being forced to adhere to single-axis movement; one cannot move diagonally in more than one axis simultaneously and, is given in the following equation:

(13) $$\begin{array}{c} \text{Distance}\;\;d=\overset{}{\underset{s_{i}\in c_{k}}{\sum }}||s_{i}-\mu_{k}||. \end{array}$$

Whenever each pair is in nonempty intersection, there exists an intersection point for the whole collection; therefore, the Manhattan distance forms an injective metric space. In the proposed optimized distance method for the given query image only the distance related cluster is searched. The optimized distance metric is given as

(14) $$\begin{array}{c} D=||\overset{n}{\underset{i=0}{\sum }}\left[{\left(R_{c}-R_{g}\right)}^{3}{+\left(G_{c}-G_{g}\right)}^{3}{+\left(B_{c}-B_{g}\right)}^{3}\right]||. \end{array}$$

The optimized method computes the cube power of the distance between the centroid and color pixel points of the three colors and determines the summation of all the added values. The modulus of the whole summed values is calculated to get the distance value.

## 4. Results and Discussion

**Figure 4:**
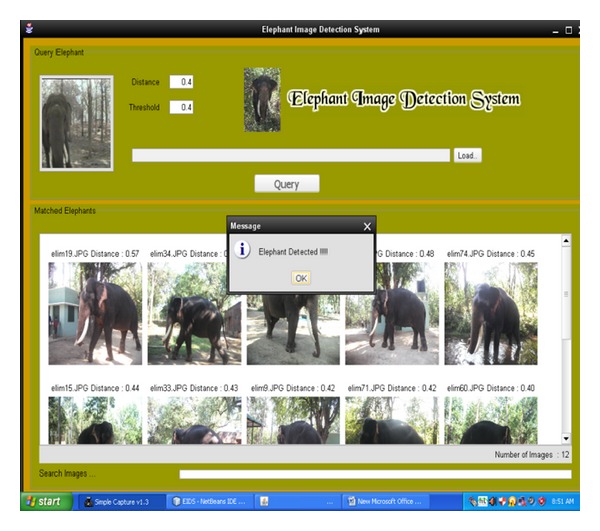
Retrieved online elephant image.

The field observations are carried out in the forest border areas in Sadivayal elephant camp. The hardware setup was arranged to capture the image of elephants. The wireless camera was mounted on a wood stick and the camera was battery powered with 12 V. Using RF receiver, the video received is converted to image frames using camcorder software in PC. The elephant image frames are stored in the PC memory and updated every 5 seconds.  Figure 4 shows the online EIDS window in which 12 elephant images are retrieved in 6.33 seconds. The elephant images are arranged in the order of similarity value obtained. As the retrieved images are more than 5, a GSM message “Elephant Detected” is sent using the AT command.

**Figure 5:**
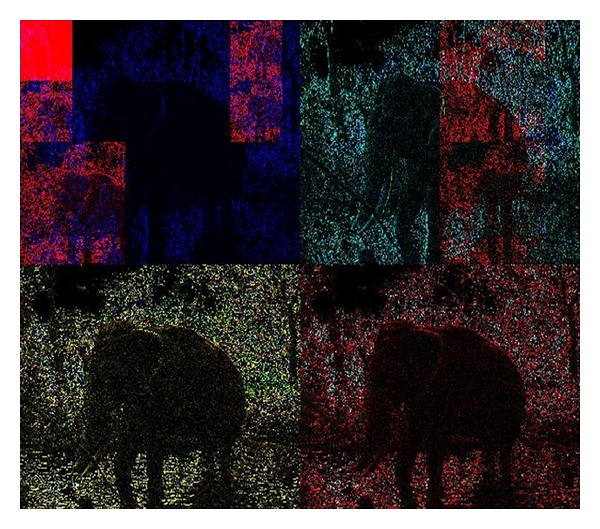
3 Level decomposed query elephant image.

**Figure 6:**
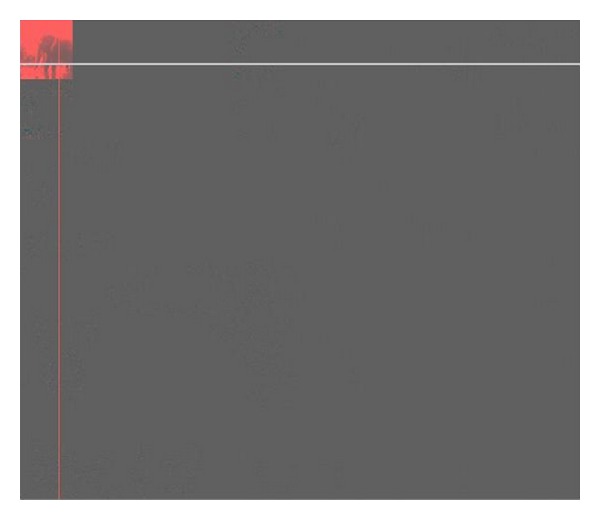
Downsampled decomposed elephant image.

In this work, we perform Haar wavelet decomposition on the raw elephant image, by determining the scaling coefficient and largest wavelet coefficients. The scaling coefficient is stored along with the difference and location (*i*, *j*) of each wavelet coefficient for every image [15]. The 3-level decomposed query elephant image is shown in Figure 5. Using the 3-level wavelet decomposition, the highest and informative elephant image features are extracted from the coefficients. These features are used during the process of the query and database image comparison of the elephants. The Haar wavelet transform of the elephant is calculated by passing it through a series of filters (high and low pass filters) and then downsampled, as we can see from Figure 6.

At each level, the elephant image is decomposed into low and high frequencies, and this decomposition halves the resolution since only half the number of samples is retained to characterize the entire image. The Haar wavelet leads to a decomposition of coefficients at level *j* in four components, and at level *j* + 1. Due to successive downsampling by 2, the image length must be a power of 2, or a multiple of a power of 2, and the length of the image determines the maximum levels into which the elephant can be decomposed.

**Figure 7:**
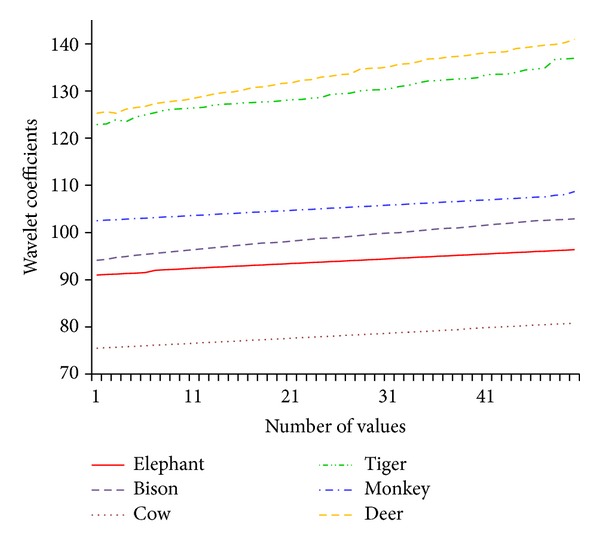
Wavelet Coefficients for Different Species.

The Haar wavelet coefficients of different species are plotted and shown in Figure 7. The Haar wavelet coefficient of each species varies with the elephant image and can be distinguished from other animals.

The value of wavelet coefficients of elephant and bison is closer because the major color is black for both species and the value lies in between 90 and 95. The coefficients for tiger and deer possess higher band in between 120 and 130. The obtained elephant coefficient is averaged to get the threshold value. We fix 0.6 as threshold value to obtain the similar elephant images from the database image for the given query image.

**Figure 8:**
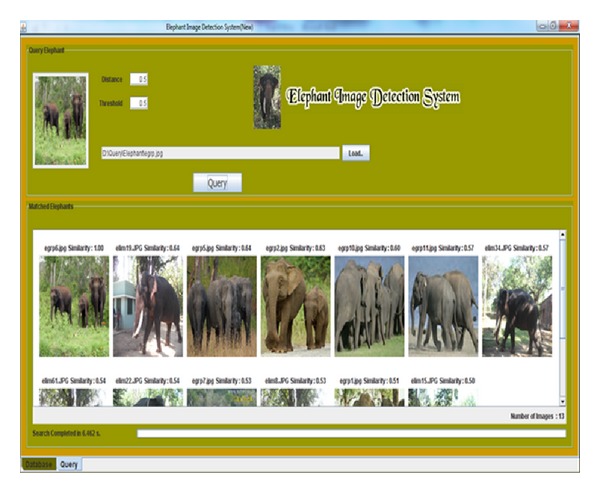
Retrieved elephant group image.

The EIDS system is tested offline with elephant and nonelephant images. All the images used in this work are in the dimensions of 3648 × 2763. We also tested the system for group of elephant images and it is shown in Figure 8.

The elephant normally moves in herds in the forest borders during the period of migration. For the given query image, the system retrieved 13 images in 6.462 seconds. We tested the system with group of elephant images of different sizes and postures. Nonelephant images like bison, bear, deer, monkey, and human which are the most commonly seen species around forest border areas are given as query, which produced zero image search result. We tested the case with a bison image as it is of similar color texture as that of elephants for which the system retrieved zero search images. On zero search result, no alert is made; hence, GSM message is not sent.

**Figure 9:**
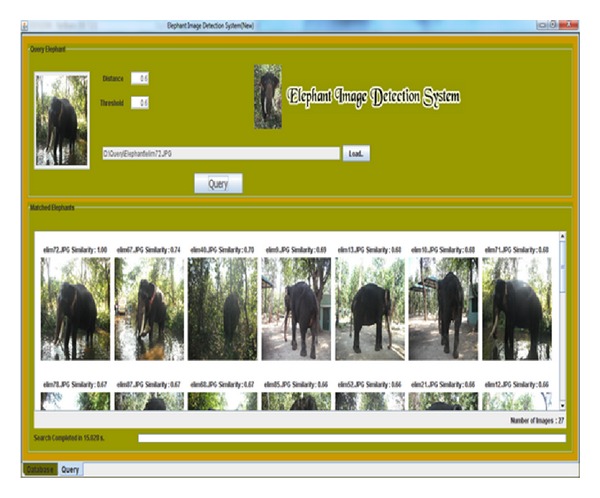
Retrieved images for optimized distance metric.

We compared the three distance measures with the number of images retrieved, retrieval time, and the retrieval rate per image. The Euclidean scheme produced 20 retrieved images with retrieval time of 21.544 seconds and the retrieval rate per image is 1.077 seconds. The Manhattan scheme produced 22 retrieved images with retrieval time of 15.067 seconds and the retrieval rate per image is 0.684 seconds. For the proposed optimized distance metric, the images retrieved were 27 as shown in Figure 9 with retrieval time of 15.028 seconds and the retrieval rate per image is 0.556 seconds.

**Figure 10:**
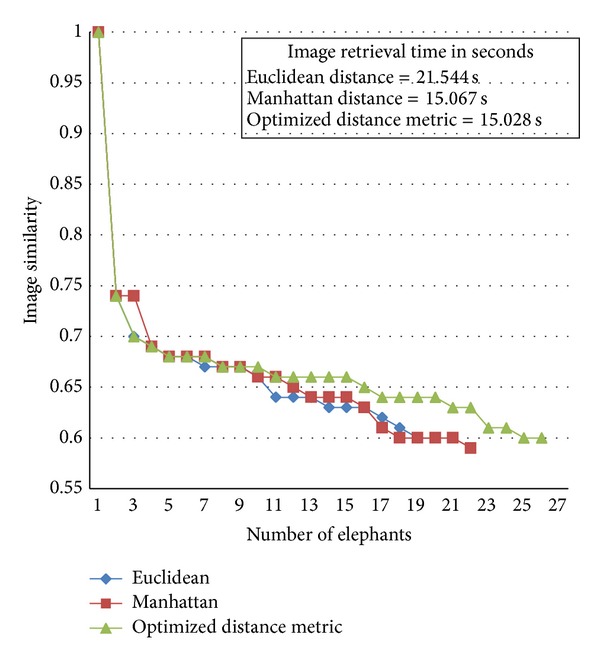
Image similarity metric comparison.

A comparison between all the distance methods is carried out in this work and the results are shown in Figure 10. It is observed that the proposed method retrieves more images with lesser time compared to the other two methods.

**Figure 11:**
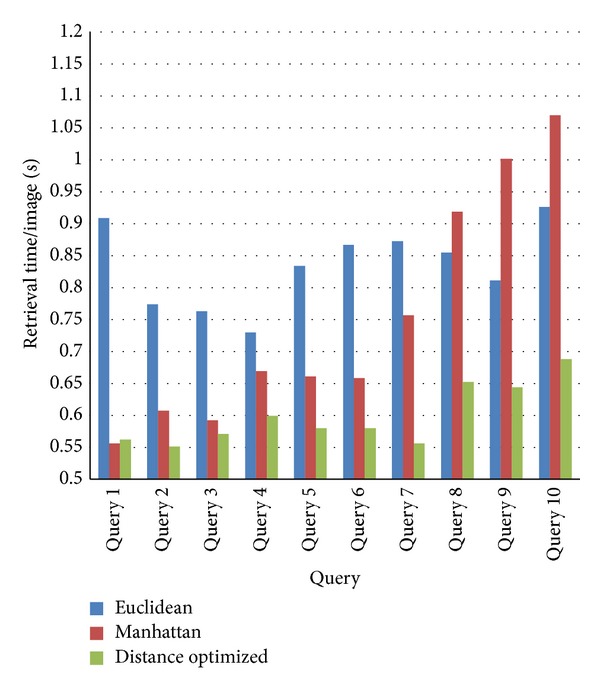
Image retrieval rate comparison.

The optimized retrieval rate improvement over another distance metric is 18%. To assess the retrieval effectiveness, 10 query elephant images are selected and tested. The average retrieval time per query elephant image is calculated for all the three distance measures and the results are shown in Figure 11. The retrieval time per image is less for the proposed distance metric compared to other methods as the metric retrieves more images which are closer to the cluster center. The images are also retrieved in lesser time interval. Due to reduction of computational time and higher image retrieval rate, the time to react for the elephant intrusion is improved using the proposed method.

The recall rate [21] is defined as the ratio of the number of relevant (same shape and position) retrieved images to the total number of images in the database

(15) $$\begin{array}{c} \text{Recall}\;\;\text{rate}=\frac{\text{Number}\;\;\text{of}\;\;\text{relevant}\;\;\text{images}\;\;\text{retrieved}\;}{\text{Total}\;\;\text{number}\;\;\text{of}\;\;\text{images}\;\;\text{in}\;\;\text{database}}. \end{array}$$

**Figure 12:**
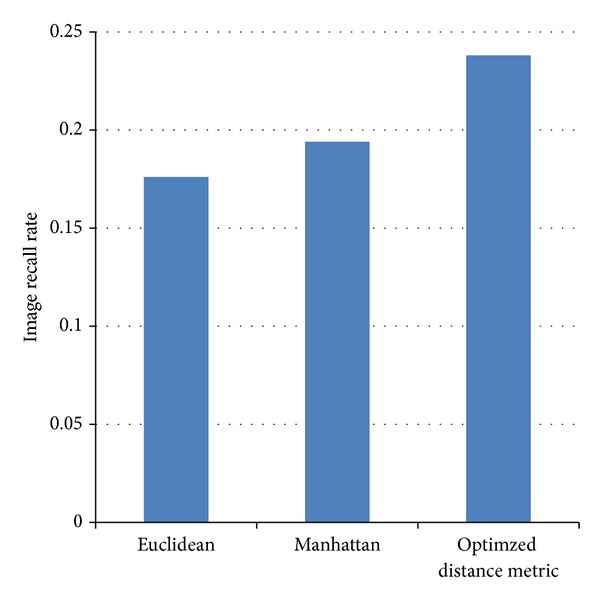
Elephant image recall rate comparison.

We compare image recall rate for the three methods as shown in Figure 12. It is observed that the optimized distance metric is 16% better than Manhattan and 18.5% better than Euclidean methods. We also tested the algorithm by varying the cluster formation.  Table 1 shows the variation of number of images retrieved, retrieval time, and retrieval rate per image.

When we fix 2 clusters, 14 images were retrieved and the retrieval time for the 14 images was found to be 10.884 and the retrieval rate per image is 0.777.

**Figure 13:**
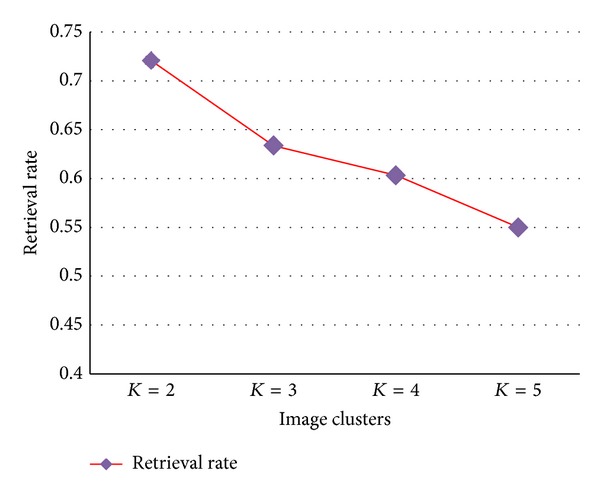
Image retrieval rate per cluster.

We varied the cluster *K* value from 2 to 5 and recorded the corresponding number of retrieved images and the retrieval rate per image from the database for the query image given. It is seen that for small number of clusters more images were retrieved and for higher number of clusters the retrieved images were less in number as shown in Figure 13. So for our analysis, we fix 2 clusters in order to retrieve more images from the database.

A comparison was made between of offline and online elephant image detection system. In the online system for the threshold values 0.4 to 0.6, the number of images retrieved varied from 5 to 12 and for offline system, it produced 17 to 22 images. This variation is due to the camera posture and image capture in frame. The required number of 5 images is achieved in online system with the proposed optimized distance method that makes the system efficient and reliable. The result comparison between the online and offline detection system is recorded in the Table 2.

## 5. Conclusion

In conclusion, the findings of our work contribute to elephant conservation issues. The work provides solutions to human-elephant conflict. The study provides insights to protect elephants from human activities and reduces the work effort of forest officials. The real time elephant identification system provides solutions to the problem of human elephant conflict and provides solution for unsupervised process of individual species identification specifically for elephants. The system is completely automated; the strength of this approach stems from the ability to narrow down the collection of potential matches in the database with the query image. Optimal results for automated identification of individual elephants are obtained with the algorithm developed and is used to rank the most likely matches, followed by final supervised visual identifications and also with an early warning sent to the forest officials about the arrival of elephants from the forest borders into the human habitat. The real time automated approach minimizes the manual work which is not possible all the time because it is difficult to monitor the presence of elephants manually when the herds march towards the forest borders. More importantly, our results demonstrate the importance of certainty in identifying approaching elephants in to human living areas and provide early warning about the elephant entry into the human habitat. We therefore recommend the use of the real time image processing technique to identify an approaching individual elephant as well as a group of elephants. The system can be implemented in forest border areas. We also solve the traits for distinguishing species where animals are differentiated on the basis of Haar wavelet color attributes. In the context of elephant conservation, the real time automated image processing system can be used for the elephant ecology learning, population monitoring, elephant habitat usage, commercial ivory poaching and human-elephant conflict. The system can also be deployed along forest border migration routes or at water holes and food plantation areas for elephant tracking and monitoring.

In this paper, we proposed and analyzed an elephant image detection system via wavelet decomposition of images, followed by feature extraction and similarity match under *F*-norm theory. We compared the retrieval performance of optimized distance metric based *K*-means clustering with the existing techniques like Euclidian distance and Manhattan distance. It turns out that optimized distance metric calculation has 18.5% improvement in recall rates. Field observations show that the proposed method can be used as an effective scheme to detect elephants in the forest border areas even in the presence of different species. This system has been rigorously tested through the various phases of the project and found to be efficient compared to the existing systems.

## Figures and Tables

**Table 1:** Database clustering for different *K* values.

*K*	Number of retrieved images	Retrieval time for images	Retrieval rate per image
2	14	10.884	0.777
3	12	7.604	0.6336
4	12	7.242	0.6032
5	9	4.949	0.5498

**Table 2:** Comparison between the online and offline observations.

Image threshold	Image distance		Retrieved images	
	Offline	Online	Offline	Online
0.4	1.0–0.49	0.57–0.36	22	12
0.5	1.0–0.49	0.44–0.34	22	6
0.6	1.0–0.59	0.47–0.38	17	5
0.7	1.0–0.74	0	2	0

## References

[1] Kumara HN, Rathnakumar S, Ananda Kumar M, Singh M (2012). Estimating Asian elephant, elephasmaximus, density through distance sampling in the tropical forests of BiligiriRangaswamy Temple Tiger Reserve, India. *Journal Tropical Conservation Science*.

[2] Sugumar SJ, Jayaparvathy R (2013). An early warning system for elephant intrusion along the forest border areas. *Current Science*.

[3] Lenin J, Sukumar R (2011). Action plan for the Mitigation of Elepant-Human conflict in India. *Final Report to the U.S. Fish and Wildlife Service*.

[4] Arivazhagan C, Ramakrishnan B (2010). Conservation perspective of Asian Elephants (*Elephasmaximus*) in Tamil Nadu, Southern India. *International Journal of Biotechnology*.

[5] Fernando P, Kumar MA, Williams AC, Wikramanayake E, Aziz T, Singh SM (2008). Review of human-elephant conflict mitigation measures practiced in South Asia. *AREAS Technical Support Document Submitted to World Bank*.

[6] King LE, Lawrence A, Douglas-Hamilton I, Vollrath F (2009). Beehive fence deters crop-raiding elephants. *African Journal of Ecology*.

[7] Loarie SR, Aarde RJV, Pimm SL (2009). Fences and artificial water affect African savannah elephant movement patterns. *Biological Conservation*.

[8] Venkataraman AB, Saandeep R, Baskaran N, Roy M, Madhivanan A, Sukumar R (2005). Using satellite telemetry to mitigate elephant-human conflict: an experiment in northern West Bengal, India. *Current Science*.

[9] Venter PJ, Hanekom JJ (2010). Automatic detection of African elephant (Loxodonta africana) infrasonic vocalisations from recordings. *Biosystems Engineering*.

[10] Vermeulen C, Lejeune P, Lisein J, Sawadogo P, Bouche P (2013). Unmanned aerial survey of elephants. *PLoS ONE*.

[11] Dabarera R, Rodrigo R Vision based elephant recognition for management and conservation.

[12] Ardovini A, Cinque L, Sangineto E (2008). Identifying elephant photos by multi-curve matching. *Pattern Recognition*.

[13] Goswami VR, Lauretta MV, Madhusudan MD, Karanth KU (2012). Optimizing individual identification and survey effort for photographic capture-recapture sampling of species with temporally variable morphological traits. *Animal Conservation*.

[14] Porwik P, Lisowska A (2005). The haar-wavelet transform in digital image processing: its status and achievements. *Machine Graphics and Vision*.

[15] Arivazhagan S, Ganesan L (2003). Texture classification using wavelet transform. *Pattern Recognition Letters*.

[16] Latha MY, Jinaga BC, Reddy VSK (2007). Content based color image retrieval via wavelet transforms. *International Journal of Computer Science and Network Security*.

[17] Manimegalai P, Thanushkodi K (2010). Content based color image retrieval using adaptive lifting. *International Journal of Advanced Networking and Applications*.

[18] Doreswamy D, Hemanth KS (2012). A novel design specification distance (DSD) based K-mean clustering performance evaluation on engineering materials' database. *International Journal of Computer Applications*.

[19] Tzortzis GF, Likas AC (2009). The global kernel *κ*-means algorithm for clustering in feature space. *IEEE Transactions on Neural Networks*.

[20] Vadivel A, Majumdar AK, Sural S Performance comparison of distance metrics in content-based image retrieval applications.

[21] Deselaers T, Keysers D, Ney H (2008). Features for image retrieval: an experimental comparison. *Information Retrieval*.

